# Malnutrition, hypertension, and hyperlipidemia as risk factors for recurrent cellulitis

**DOI:** 10.1016/j.jvscit.2021.02.017

**Published:** 2021-05-03

**Authors:** Yurie Norimatsu, Yuta Norimatsu

**Affiliations:** Department of Dermatology, Ageo Daiichi Clinic for Internal Medicine and Pediatrics, Ageo City, Japan; Department of Dermatology, Ageo Daiichi Clinic for Internal Medicine and Pediatrics, Ageo City, Japan; Department of Dermatology, University of Tokyo Graduate School of Medicine, Tokyo, Japan

Yoshida et al^1^ reported a case of recurrent cellulitis that was treated via lymphovenous anastomosis (LVA). Lymphedema, along with chronic venous insufficiency, peripheral circulatory disturbance, and deep vein thrombosis, are risk factors for recurrent cellulitis.^2^ Compression therapy has been increasingly gathering attention as a treatment of lymphedema and is known to prevent recurrence.^3^ In addition, LVA, as a treatment of lymphedema, might also prevent the recurrence of cellulitis. In contrast, Norimatsu and Ohno^4^ reported that hyperlipidemia, hypertension, hypoalbuminemia, and lymphedema are significant risk factors for recurrent cellulitis in the Japanese population. Hypoalbuminemia, in particular, is a risk factor in the Japanese population. Moreover, the body mass index of Japanese patients with cellulitis will not be as high as that of patients in other countries, suggesting that poor nutrition might be involved in the recurrence of cellulitis.^4^ Yoshida et al^1^ did not report the serum albumin levels or body mass index; therefore, their patient's nutritional status is unclear. Patients with dementia and those with cancer are known to have a greater risk of malnutrition.^5^^,^^6^ Thus, the risk of their patient being malnourished was high owing to the medical history of dementia and cervical cancer. Therefore, although the lymphedema in their patient was treated with LVA, their patient's nutritional status could have been an unresolved risk factor for cellulitis. Careful monitoring and optimization of the patient's nutritional status is, therefore, also required to help prevent the future recurrence of cellulitis.

Hypertension is also a risk factor for the recurrence of cellulitis, independently of the presence of lymphedema.^4^ In contrast, it is unclear why hyperlipidemia has been associated with the recurrence of cellulitis in the Japanese population.^4^ Oxidized low-density lipoprotein is known to activate dendritic cells (DCs) in the presence of hyperlipidemia.^7^ Yoshida et al^1^ also attributed activated DCs to the recurrence of cellulitis in patients with lymphedema. DCs might have also played an important role because they are involved in both hyperlipidemia and lymphedema, and it is unknown whether their patient had had hypertension or hyperlipidemia.

In conclusion, malnutrition, hypertension, and hyperlipidemia are as important as lymphedema in Japanese patients with recurrent cellulitis.
